# Do standard optometric measures predict binocular coordination during reading?

**DOI:** 10.16910/jemr.13.6.6

**Published:** 2021-01-21

**Authors:** Joëlle Joss, Stephanie Jainta

**Affiliations:** Institute of Optometry, University of Applied Sciences, Northwestern Switzerland

**Keywords:** eye movement, eye tracking, reading, individual differences, binocular coordination, heterophoria, saccade disconjugacy, vergence drift, fixation disparity

## Abstract

In reading, binocular eye movements are required for optimal visual processing and thus, in case of asthenopia or reading problems, standard orthoptic and optometric routines check individual binocular vision by a variety of tests. The present study therefore examines the predictive value of such standard measures of heterophoria, accommodative and vergence facility, AC/A-ratio, NPC and symptoms for binocular coordination parameters during read-ing. Binocular eye movements were recorded (EyeLink II) for 65 volunteers during a typical reading task and linear regression analyses related all parameters of binocular coordination to all above-mentioned optometric measures: while saccade disconjugacy was weakly pre-dicted by vergence facility (15% explained variance), vergence facility, AC/A and symp-toms scores predicted vergence drift (31%). Heterophoria, vergence facility and NPC ex-plained 31% of fixation disparity and first fixation duration showed minor relations to symp-toms (18%). In sum, we found only weak to moderate relationships, with expected, selective associations: dynamic parameter related to optometric tests addressing vergence dynamics, whereas the static parameter (fixation disparity) related mainly to heterophoria. Most sur-prisingly, symptoms were only loosely related to vergence drift and fixation duration, re-flecting associations to a dynamic aspect of binocular eye movements in reading and poten-tially non-specific, overall but slight reading deficiency. Thus, the efficiency of optometric tests to predict binocular coordination during reading was low – questioning a simple, straightforward extrapolation of such test results to an overlearned, complex task.

## Introduction

Up-to-date, we know exactly, how the horizontal binocular coordination during reading typically works: the movements of the left and right eye during each saccade are not identical, leading to a disconjugacy during the saccade (1, 2). This disconjugacy is followed by a vergence drift during the fixation which (partly) corrects the disconjugacy (2, 3, 4, 5, 6, 7) and actively maximizes the overlap of both retinal images (8, 9). In reading English or German texts, saccades are mainly performed from left to right and sometimes from right to left (i.e. regressions) and we basically observe horizontal disconjugacy during these saccades of about 0.2 degrees (10, 11) and vergence drifts of about 0.1 degrees, on average (9, 11). Additionally, in most fixations a so-called fixation disparity remains at the end of reading fixations, which shows a pronounced horizontal and only small vertical component (9). A horizontal fixation disparity reflects, that the visual axes of the eyes cross slightly in front (eso) or behind (exo) the plane of fixation (12, 13, 14). This small vergence error typically amounts to about one character size, that is, 0.2 to 0.5 degrees (15, 16) and a single, stable image of the text is still perceived due to Panums’ fusional area (14).

Technical instrumentation allows for very precise objective measurement of the individual oculomotor behavior (17, 18, 19). In clinical optometric and ophthalmologic settings, however, methods that need charts, an experienced examiner and which rely mostly on the answers and perceptions of the patient are usually applied to assess individual binocular status (20, 21, 22, 23). In this context, several parameters are required to qualify a patient’s individual binocular status: based on visual acuity and refractive measures, heterophoria, vergence and accommodative parameters and asthenopic symptoms need to be assessed. Horizontal heterophoria is a classical, quantifiable, clinical parameter of vergence at rest, reflecting a status of no vergence demand, which is stabilized by accommodation inputs to vergence and proximity cues (12, 24, 25). Prominent subjective tests include, for example, the Maddox-Wing test (for details, see Pointer (26)) in anglo-american settings and the “Measurement and Correction Methodology after H.-J. Haase” (MCH) in German speaking countries (22). Vergence and accommodative parameters are usually assessed with vergence and accommodative facility tests, positive and negative fusional reserves, accommodative amplitude and a calculation of the accommodative convergence to accommodation ratio (AC/A-ratio); further tests, such as the near point of convergence and the assessment of stereopsis are also part of a routine optometric oculomotor examination and give additional information about the vergence system (25). Finally, the assessment of asthenopic symptoms is of central importance (25): standardized questionnaires like the Convergence Insufficiency Symptom Survey (CISS) sum up symptoms along different aspects of asthenopia (27). In sum, clinical optometric tests to characterize the individual binocular status are time consuming and call for experienced examiners. Surprisingly, only few studies show clear relations between standard optometric measures and binocular coordination during reading or reading performance in general (28): recent reports showed a shift of fixation disparity towards eso in reading when participants showed lower vergence facility (29). Some studies also related heterophoria to single parameters of binocular coordination in reading, showing a reduction in binocular advantage with increasing heterophoria (18) and larger saccade disconjugacy for larger exophoria (30), for example. Some studies assessed optometric measures and binocular parameters during reading and observed the reaction on training (31). But there are various studies assessing optometric measures or the training of binocular parameters and – theoretically – relating them to binocular coordination used for reading without objectively measuring it (32, 33, 34, 35, 36). However, some of these studies addressed reading performance taking reading times into account (34) or others related eye movement parameters to optometric parameters (37, 38). Furthermore, several studies in the last decades set out to show, which optometric measures best predicted asthenopic symptoms and suggested subjective fixation disparity as relevant predictor (39, 40). However, even though objective and subjective fixation disparity are correlated, they show a different overall pattern in reaction to prisms or training, for example (41, 42), and thus, the question remains, whether objective fixation disparity is also a good predictor for asthenopic symptoms.

Thus, it is highly timely to collect typical optometric data to characterize the individual binocular status (heterophoria, vergence and accommodative facility, near point of convergence, AC/A-ratio and asthenopic symptoms) and relate these to aspects of the binocular coordination during reading (disconjugacy during saccades, vergence drift, objective fixation disparity and first fixation duration). To reiterate, we know a lot about the physiology of single optometric tests for binocular vision (22, 25) and we know the physiology of binocular eye movements during reading (28) – and yet, the predictive value of a single (or multiple) standard optometric test in this context is still missing. As soon as best predictors for binocular coordination during reading are identified, individual prescriptions in the day-to-day practice can potentially be optimized and training or treatment effects are easily shown in real reading tasks.

## Methods

### Participants

In total, 65 young volunteers (35 female and 30 male) aged 18 to 39 years (M = 24.9, SD = 3.6 years) participated, reporting German as native language, no dyslexia, no former or actual ocular pathologies (e.g. strabismus) or surgery (e.g. corneal surgeries) (28). Every participant underwent a thorough optometric examination, which was similar to other registered randomized clinical trials (43, 44): all participants had a monocular uncorrected visual acuity of 0.8 or better (in decimal units) at a viewing distance of 60 cm for each eye (60 cm corresponds to the experimental viewing distance); right eye spherical equivalent ranged between -2.13 and 1.38 dpt (M = 0.04, SD = 0.63) and left eye spherical equivalent ranged between ‑2.00 and 0.88 dpt (M = 0.05, SD = 0.54) for far testing (subjective and objective). Stereo acuity thresholds were 100’’ or better and 65% of our participants showed a right eye dominance. Further, participants did not show strabismic eye deviations, vertical heterophoria greater than 1 pdpt or wear prismatic corrections.

### Reading task and eye movement recordings

Participants silently read 20 sentences from the Potsdam-Sentence-Corpus (PSC; see Kliegl, Nuthmann (45)). Sentences were presented in 4 blocks of 5 sentences at 60 cm reading distance. We selected sentences containing 8 to 13 words, and they differed in total length from 55 to 75 characters. One character corresponded to approximately 0.29 degrees of horizontal visual angle. Sentences were presented in black, Courier New font size 12, on a white background (24 cd/m2, surrounding room lighting: 127 lux). In one-third of all reading presentations, a multiple-choice question about the content of the sentence was presented, to ensure reading for meaning. Two participants who showed more than 10% incorrect responses were excluded from data analysis.

**Figure 1.**
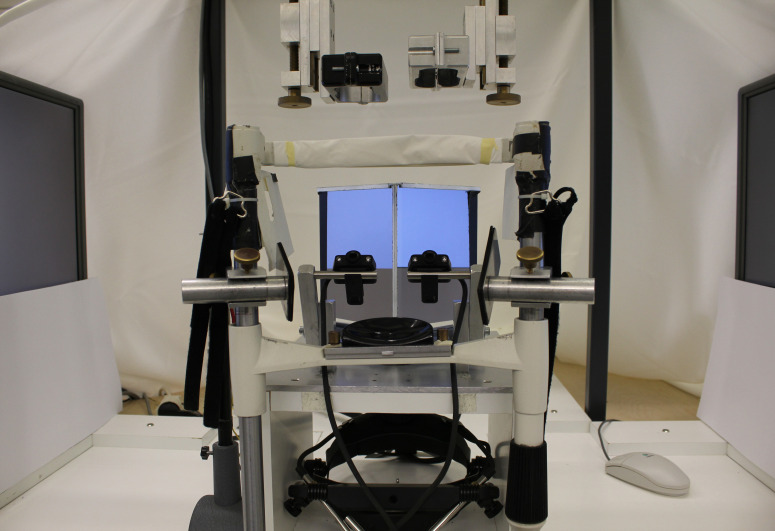
Picture of the experimental set-up. Two mirrors in the centre reflect the image of each screen to one eye only. The cameras of the Eyelink II system were positioned as close as possible to the participants’ eyes to enable maximal measurement resolution and field view.

Binocular eye movements were recorded during all sentence presentations with the video-based eye tracker EyeLink II (details provided by SR Research Ltd, Osgoode ON, Canada; 500 Hz sampling frequency). The experimental set-up (as shown in Figure 1) has been described in detail elsewhere (10, 18, 30, 46, 47, 48), but key aspects were the following: all calibrations were run monocularly, using a 3-point calibration grid (±5 degrees) and repeated prior to each reading block (i.e. every 5 sentences); pupil size variations were further measured and checked for co-variation. We then calculated the version signal from both single eye recordings, i.e. the conjugate eye movement [(left eye+right eye)/2], and extracted saccades and fixations in reading (16, 18, 46, 49). Next, we calculated the vergence signal, that is, the disconjugate eye movement [left eye–right eye]. The difference in vergence between the beginning and the end of a saccade was calculated as the saccade disconjugacy (arcmin). We then calculated the vergence drift (arcmin) in vergence occurring during the fixation period, corresponding to the change in vergence between the beginning and the end of the fixation period. Objective fixation disparity (arcmin) at the end of each fixation was then defined and calculated as the difference between the measured vergence angle and the geometrically expected vergence angle (for text presented at 60 cm). We extracted 190 (SD = 36) saccades and adjacent fixations per participant on average and pooled these data to provide an estimation of the individual binocular coordination during reading.

Additionally, to control for typical reading performance we also calculated the average first fixation duration for each participant (58).

### Measurements of horizontal heterophoria

(1) The Maddox-Wing test (Trusetal Verbandstoffwerk GmbH, Germany) was used at 30 cm under full dissociation of the visual stimuli (for details see Pointer (26) and (50)). The right eye only saw an arrow, while the left eye saw a numbered scale. The participant reported at which number the arrow points, which represented the heterophoria in pdpt.

(2) Horizontal heterophoria was also measured at 6 m following the “Guidelines for the Application of the Measuring and Correcting Methodology after H.-J. Haase” (MCH; see www.ivbs.org for details). The test stimuli are presented monocularly (by polarization) under peripheral (or partially central) fusion. Prisms are placed before the participant’s eyes until the dissociated parts of the test stimuli are subjectively perceived as aligned (22). The value of prisms needed for this alignment represented the heterophoria in pdpt.

(3) Objective horizontal heterophoria was measured at 60 cm with the EyeLink II (51): the participants fixated a central binocular cross for 2.5 s, followed by another cross which was presented to one eye only (for 15 s). Then again, the binocular target was presented for another 2.5 s, followed by a 15 s monocular target to the fellow eye. For each pair of binocular-monocular fixation, objective heterophoria was calculated as difference between vergence angle at the end of monocular fixation minus vergence angle at the end of binocular fixation (given in degree) (51).

### Measures of vergence and binocular accommodative facility

We used a vergence facility prism (12 base-out and 3 base-in) placed in front of the participants eyes; the participant fixated on a vertical row of letters (size corresponding to visual acuity of 1.0) at 40 cm and reported when the letters were single and clear. The lenses were alternated during 60 s (“flipped”) and the number of cycle (2 flips) per minute (cpm) was counted (25). Binocular accommodative facility was measured similarly, however, using ± 2.00 dpt binocular lenses (Bernell accommodative flipper, item number: BC1270+). Again, the participant reported when the letters were single and clear. The number of cycles was counted during 60 s (25).

### Measurement of AC/A-ratio and near point of convergence (NPC)

AC/A-ratio was measured using the Maddox-Wing test (at 30 cm) by determining the dissociated heterophoria at baseline and with +1.50 dpt and ‑1.50 dpt lenses placed in front of the participant’s eyes. The differences in vergence (pdpt) from baseline to +1.50 dpt and ‑1.50 dpt were averaged and divided by 3 dpt. The near point of convergence (NPC) was measured with the participant fixating the tip of a pencil, which was approached towards the eyes until the participant reported the tip to become double or when the examiner saw that one eye drifted away. The distance from the break point was then measured to the bridge of the nose (43).

### Assessment of asthenopic symptoms

All participants worked through a German version of the CISS questionnaire (convergence insufficiency syndrome survey), which consists of 15 questions about eye- and vision related symptoms during near tasks (25). The total sum gave the symptom score (52).

### Data selection and statistical analysis

To facilitate comparison, all values of binocular coordination and heterophoria were converted into degrees: objective measures (arcmin) were divided by 60 and heterophoria measures (pdpt) were multiplied by 0.57 (i.e. arctan (0.01 m/ 1 m)). All variables were then tested for normal distribution (Shapiro-Wilks test), transformed (if necessary) and centered before performing linear regression analyses (least-square fit and standard evaluation of coefficient estimations), using SPSS® Statistics Version 25.0.0.2 (IBM® Corporation, Armonk, 2018). Note, that we only entered 6 main effects (heterophoria, vergence and binocular accommodative facility, AC/A-ratio, NPC and asthenopic symptoms) as continuous variables into our regression analysis, to avoid model overfitting. We ran a set of four regression models (R1-4) to predict:

R1. saccade disconjugacy

R2. vergence drift

R3. objective fixation disparity

R4. reading first fixation duration

This set of regressions was repeated 3 times, since we changed the measure of heterophoria: we used Maddox-Wing heterophoria (R1a, R2a, R3a, R4a), MCH heterophoria (R1b, R2b, R3b, R4b) and objective heterophoria (R1c, R2c, R3c, R4c) in separate regressions.

Please note that we also ran all analyses considering only heterophoria size (regardless of exo or eso deviation) resulting in an almost identical pattern of results.

## Results

### Binocular coordination during reading

**Figure 2.**
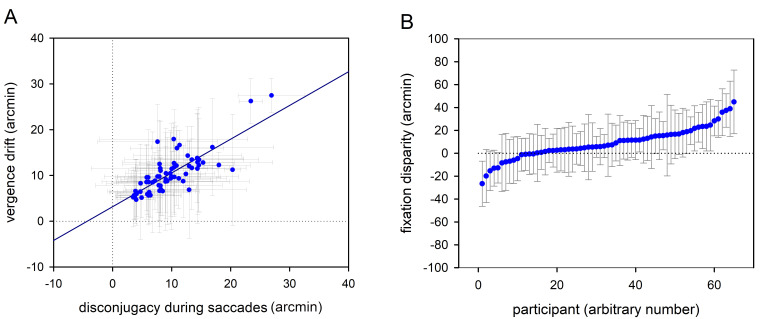
Binocular coordination during reading: Scatterplots showing (A) the correlation between disconjugacy during saccades and vergence drift during fixations and (B) mean fixation disparities for all participants. Exo fixation disparity is shown by negative values, while eso fixation disparity is reflected by positive values. Dots reflect mean values (arcmin) and grey whiskers show standard deviations (arcmin). The horizontal and vertical dotted lines mark zero values.

Average sentence reading times for binocular reading was 2.14 s (SD = 0.6) and mean fixation duration ranged between 200 and 350 ms (M = 254, SD = 27). Further, average forward (left to right) saccade amplitude was 2.27 deg (SD = 0.42) and thus, all observed parameters reflected typical reading behaviour (31, 49, 53). Disconjugacy during saccades ranged between 3 and 28 arcmin (M = 10, SD = 5) and vergence drifts ranged between 0.1 and 20 arcmin (M = 7, SD = 4). We observed a typical pattern of binocular coordination during reading: disconjugacy and drift correlated well (r = 0.53; p < 0.001; see Figure 2A) and horizontal fixation disparities ranged between 21 and 42 arcmin (M = 12, SD = 13), with a majority of participants showing an eso fixation disparity (see Figure 2B).

### Measures of binocular status

On average, Maddox-Wing-heterophoria (med = ‑0.57 deg, IQR = 2.15) and objective heterophoria (M = ‑1.77 deg, SD = 1.19) gave negative values, i.e. they showed an exophoria (see Table 1). In contrast, MCH heterophoria (med = 0.86 deg, IQR = 3.51) was positive, i.e. it corresponded to an esophoria. Comparing all heterophoria tests (using Bonferroni-corrected significance level) showed that MCH heterophoria significantly differed from Maddox heterophoria (Z = 5.57, p < (0.001/3)) and objective heterophoria (Z = 6.59, p < (0.001/3)), while Maddox heterophoria was also significantly larger than objective heterophoria (Z = ‑3.52, p < (0.01/3)).

**Table 1.** Mean (M) values, standard deviation (SD), median, interquartile ranges (IQR), minimum and maximum values of Maddox-Wing, MCH and objective heterophoria (n = 65). Negative values correspond to exophoria, and positive values to esophoria.

	Maddox-Wing heterophoria		MCH heterophoria		Objective heterophoria	
	deg	pdpt	deg	pdpt	deg
M	-1.21	-2.11	1.10	1.92	-1.77
SD	1.83	3.20	2.75	4.80	1.19
Median	-0.57	-0.99	0.86	1.50	-1.75
IQR	2.15	3.75	3.51	6.13	1.68
Min	-5.73	-10.00	-4.30	-7.50	-5.50
Max	3.44	6.00	8.59	15.00	0.47

Vergence facilities (measured with 12 base-out and 3 base-in) ranged between 5 and 18 cpm (M = 11.4, SD = 3.4), while binocular accommodative facility ranged between 1 and 17 cpm (M = 6.5, SD = 4.0). Next, AC/A-ratio (measured at 30 cm with ±1.50 dpt ) ranged between 0.7 and 4.3 (M = 2.3, SD = 0.7) and the near point of convergence ranged between 3 and 14 cm (M = 6.1, SD = 3.0). Finally, the CISS-score (25) ranged between 0 and 32 points (M = 14.7, SD = 7.4). All values are presented in Table 2.

**Table 2.** Overview of independent optometric variables used for the first set of linear regression models: vergence facility (VF; 12 BO and 3 BI during 60 s at 40 cm), binocular accommodative facility (AF; ±2.00 dpt flipper during 60 s at 40 cm), AC/A-ratio (with ±1.50 dpt at 30 cm), near point of convergence (NPC) and asthenopic symptoms (CISS questionnaire score). Test-for-normality results are also shown (Shapiro-Wilks).

	VF	AF		AC/A	NPC		Symptoms	
	cpm	cpm			cm			
M	-1.21	-2.11		1.10	1.92		-1.77	
SD	1.83	3.20		2.75	4.80		1.19	
Median	-0.57	-0.99		0.86	1.50		-1.75	
IQR	2.15	3.75		3.51	6.13		1.68	
Min	-5.73	-10.00		-4.30	-7.50		-5.50	
Max	3.44	6.00		8.59	15.00		0.47	
Shapiro-Wilks (W)	0.97	0.93	**	0.98	0.88	***	0.96	*

Note. *: p ≤ 0.05; **: p ≤ 0.01; ***: p ≤ 0.001.

### Regression analysis: predicting binocular coordination during reading

Disconjugacy during saccades (R1), vergence drift (R2), objective fixation disparity (R3) and first fixation duration (R4) during reading were predicted by heterophoria (Maddox-Wing, MCH and objective measurements), vergence and accommodative facility, AC/A-ratio, near point of convergence and asthenopic symptoms:

R1. To predict individual disconjugacy during reading saccades only vergence facility showed a significant effect, while all other parameters remained non-significant (all t-values <1). When Maddox-Wing heterophoria was entered into the regression model (R1a), vergence facility (b = 0.096; SE = 0.0043; p = 0.03) explained about 15% of variance. Entering MCH heterophoria (R1b) yield about the same effect for vergence facility (b = 0.0092; SE = 0.0043; p = 0.03) and explained about 14% of variance. Finally, when heterophoria was measured using objective eye tracking data (R1c), again only vergence facility (b = 0.0101, SE = 0.0044, p = 0.02) showed a significant effect, explaining about 15% of variance, the highest amount in our analysis set (see Table 3).

R2. The individual vergence drift during reading could be significantly predicted by vergence facility and AC/A-ratio, while all other parameters remained non-significant. When Maddox-Wing heterophoria was used in the regression model (R2a), vergence facility (b = 0.0079, SE = 0.0029, p = 0.01) and AC/A-ratio (b = -0.0460, SE = 0.0119, p ≤ 0.001) explained about 29% of variance. Next, when MCH heterophoria was entered into the model (R2b), similar effects were observed for vergence facility (b = 0.0070, SE = 0.0029, p = 0.02) and AC/A-ratio (b = ‑0.0383, SE = 0.0107, p ≤ 0.001) explaining 27% of variance. More importantly, when entering objective heterophoria (R2c) similar effects were observed for vergence facility (b = 0.0084, SE = 0.0029, p = 0.01) and AC/A-ratio (b = -0.0441, SE = 0.0110, p ≤ 0.001), but here also the symptoms score (b = 0.0163, SE = 0.0077, p = 0.04) became significant. All three variables explained about 31% of variance (see Table 3).

R3. The individual fixation disparity could be predicted by heterophoria, vergence facility and near point of convergence, while all other parameters remained non-significant. When Maddox-Wing heterophoria was entered into the regression model (R3a), Maddox-Wing heterophoria (b = 0.0650, SE = 0.0173, p ≤ 0.001), vergence facility (b = 0.0241, SE = 0.0098, p = 0.02) and near point of convergence (b = 0.0202, SE = 0.0097, p = 0.04) explained 24% of variance. Next, when entering MCH heterophoria (R3b), only MCH heterophoria (b = 0.0205, SE = 0.0100, p = 0.05) explained 12% of variance (p = 0.25). And finally, when objective heterophoria was added to the model (R3c), objective heterophoria (b = 0.1100, SE = 0.0241, p ≤ 0.001), vergence facility (b = 0.0277, SE = 0.0095, p ≤ 0.01) and near point of convergence (b = 0.0211, SE = 0.0091, p = 0.02) explained 31% of variance (see Table 3).

**Table 3.** Regression analyses predicting parameters of binocular coordination during reading by heterophoria, vergence and accommodative facility, AC/A-ratio, near point of convergence (NPC) and asthenopic symptoms for a sample of N=65.

	R1c: Disconjugacy			
Fixed factor	b	SE	t	
(Intercept)	0	0.0111	0	
Objective heterophoria [deg]	0.0086	0.0111	0.78	
Vergence facility [cpm]	0.0100	0.0044	**2.31**	*
Acc. facility [cpm^0.5^]	-0.0021	0.0186	-0.11	
AC/A	0.0081	0.0167	0.49	
NPC [cm]	-0.0010	0.0042	-0.25	
Symptoms [points^0.5^]	0.0032	0.0117	0.27	
	R2c: Vergence drift			
Fixed factor	b	SE	t	
(Intercept)	0	0.0073	0	
Objective heterophoria [deg]	0.0129	0.0074	1.75	
Vergence facility [cpm]	0.0084	0.0029	**2.90**	**
Acc. facility [cpm^0.5^]	-0.0229	0.0123	-1.86	
AC/A	-0.0441	0.0110	**-4.00**	***
NPC [cm]	0.0002	0.0028	-0.05	
Symptoms [points^0.5^]	0.0163	0.0077	**2.11**	*
	R3c: Obj. fixation disparity			
Fixed factor	b	SE	t	
(Intercept)	0	0.0240	0	
Objective heterophoria [deg]	0.1100	0.0241	**4.56**	***
Vergence facility [cpm]	0.0277	0.0095	**2.93**	**
Acc. facility [cpm^0.5^]	-0.0640	0.0404	-1.59	
AC/A	-0.0498	0.0361	-1.38	
NPC [cm]	0.0211	0.0091	**2.32**	*
Symptoms [points^0.5^]	-0.0070	0.0253	-0.28	
	R4c: Fixation duration			
Fixed factor	b	SE	t	
(Intercept)	0	3.1451	0	
Objective heterophoria [deg]	-2.3868	3.1579	-0.76	
Vergence facility [cpm]	0.2493	1.2368	0.20	
Acc. facility [cpm^0.5^]	-2.3020	5.2864	-0.44	
AC/A	3.9986	4.7248	0.85	
NPC [cm]	1.5724	1.1925	1.32	
Symptoms [points^0.5^]	8.0425	3.3127	**2.43**	*

Note. *: p ≤ 0.05; **: p ≤ 0.01, ***: p ≤ 0.001

R4. The individual first fixation duration could be predicted by asthenopic symptoms only, while all other parameters remained non-significant. When Maddox-Wing heterophoria was entered into the regression model (R4a), asthenopic symptoms (b = 8.2970, SE = 3.3305, p = 0.016) explained 17% of variance. Next, when entering MCH heterophoria (R4b), asthenopic symptoms (b = 8.3221, SE = 3.2924, p = 0.014) explained 19% of variance (p = 0.05); when objective heterophoria was added to the model (R4c), asthenopic symptoms (b = 8.0425, SE = 3.3127, p = 0.018) explained 18% of variance (see Table 3).

### Exploratory data analysis regarding asthenopic symptoms

Since symptoms are an important, clinical criterion in the assessment of binocular problems, we further explored the data and showed which objective (R5) and optometric measures (R6; see table 4) best predict asthenopic symptoms:

R5. When predicting asthenopic symptoms by objective parameters of binocular coordination, again only vergence drift and fixation duration showed a significant impact: vergence drift (b = 4.6936, SE = 1.9672, p = 0.02) and fixation duration (b = 0.0136, SE = 0.0045, p ≤ 0.01) explained about 19% of variance in asthenopic complaints (see Table 4, upper part, named R5).

R6. Further, when asthenopic symptoms were predicted by subjective, optometric measures, none showed any significant contribution and the total amount of explained variance (by all measures) ranged below 1 % (see Table 4; lower part, named R6).

**Table 4.** Regression analyses predicting asthenopic symptoms by objective parameters (objective heterophoria, vergence drift, saccade disconjugacy, objective fixation disparity and fixation duration) and subjective optometric measures (Maddox-Wing heterophoria, vergence and accommodative facility, AC/A-ratio and near point of convergence (NPC) for a sample of N=65.

	R5: Asthenopic symptoms			
Fixed factor	b	SE	t	
(Intercept)	0	0.1130	0	
Objective heterophoria [deg]	0.0356	0.1066	0.33	
Vergence drift [deg]	4.6936	1.9672	**2.39**	*
Disconjugacy [deg]	-0. 6838	1.4200	-0.48	
Obj. fixation disparity [deg]	-0.7151	0.5915	-1.21	
Fixation duration [ms]	0.0136	0.0045	**3.04**	**
	R6: Asthenopic symptoms			
Fixed factor	b	SE	t	
(Intercept)	0	0.1234	0	
Maddox heterophoria [deg]	0.0545	0.0848	0.64	
Vergence facility [cpm]	-0.0356	0.0477	-0.75	
Acc. facility [cpm^0.5^]	0.0589	0.2014	0.29	
AC/A	-0.0579	0.1988	-0.29	
NPC [cm]	0.0456	0.0473	0.96	

Note. *: p ≤ 0.05; **: p ≤ 0.01, ***: p ≤ 0.001

## Discussion

In the present study, we replicated the stereotyped pattern of binocular eye movements during reading: disconjugacies during saccades are followed by a drift during fixations and small vergence errors remained at the end of fixations (2, 12), while reading times, numbers of saccades and saccade amplitudes, all resembled a typical eye movement behavior during reading (49, 53). Furthermore, we found a typical range of binocular vision qualities in our participant sample, showing heterophorias between 6 (exo) and 8 (eso) degrees, vergence facilities between 6 and 18 cpm, binocular accommodative facilities ranging from 1 to 17 cpm, near point of convergence amounting to 6.1 cm, on average, and asthenopic symptoms ranging up to 32 points of CISS score.

While all these variables showed typical mean values and ranges, the measured AC/A-ratios ranged between 0.7 and 4.3 corresponding to a typical AC/A spans but reflecting slightly lower average values (25).

When using optometric tests to predict single aspects of binocular coordination during reading, we observed very specific relations: While vergence facility predicted saccade disconjugacy, drifts during fixations were related to vergence facility, AC/A and symptoms score. This observation fits our current understanding, as well as previous studies (31, 32): saccade disconjugacy has been shown to be related to dyslexic problems and asthenopic complaints. But it is also very important to note that these optometric tests could only explain about 15% of disconjugacy in this present study and only about 30% of vergence drifts, thus reflecting only weak to modest relationships between optometric tests and dynamic aspects of binocular coordination during reading. Next, fixation disparities during reading were best predicted by heterophoria, vergence facility and near point of convergence. This observation is also in line with previous studies (29, 54, 55) and reports of associations of asthenopia and fixation disparity in non-reading tasks (56). Taking into account that all heterophoria tests were applied at different viewing distances, we nevertheless found highest predictive values for objective heterophoria measures; this in in line with previous results for other viewing tasks (54). Finally, reading fixation duration was related to symptoms score only. This is in line with previous studies which found no significant effect of binocular coordination aspects on fixation duration (18, 28, 34); but opposed to Jainta, Jaschinski (48), who found an increase of fixation duration for poor vergence adjustments. The relation between objectively measured vergence drift and fixation duration and asthenopic symptoms was further confirmed when objective measures only predicted asthenopic symptoms in this present study. This reflects that asthenopic symptoms could be related to longer fixation durations, i.e. slower reading. More interestingly, no subjective (i.e. optometric) measure explained any variance in symptoms, neither as a single measure nor when all measures were statistically combined (less than 1% explained variance). Please note here, that the CISS score has been discussed to be a statistically fragile measurement, probably due to its nature as questionnaire and its corresponding individual, multifaceted interpretation (44, 57). It is still unclear whether symptoms influence the reading process, i.e. lead to longer fixation duration, or if the longer fixation duration cause asthenopic complaints.

Finally, all optometric test measures (heterophoria, vergence and accommodative facilities, AC/A-ratio and NPC), as well as all aspects of binocular coordination during reading (disconjugacy, drift and fixation disparity) did not further relate to first fixation duration in reading (all correlation coefficients <0.2, explaining less than 5% of variance). This observation is in line with previous reports, showing that fixation duration and overall reading times are mainly driven by cognitive processes (58) and are not specifically linked to binocular coordination (18, 28, 34, 36). Note here that our present study did not test longer reading sessions or participants with severe binocular impairments.

In sum, we showed that there is a selective value in optometric tests of binocular qualities when predicting binocular eye movements during reading: tests for vergence dynamics and accommodative inputs related to saccade disconjugacy and vergence drifts during reading. Heterophoria measures together with the near point of convergence and vergence facility related to fixation disparity during reading. More importantly, asthenopic complaints were only related to vergence drifts and fixation duration. But still, a large part of variance is not explained. Best predictions for fixation disparities during reading ranged up to 30% of explained variance, leaving a substantial amount of physiological variance unexplained; these observations critically question the usefulness of optometric tests to predict binocular coordination during reading. Further research on longer reading sessions or disruptive effects of more severe binocular conditions is clearly needed to finally evaluate standard optometric testing in respect to extrapolations to day-to-day reading situations. Nevertheless, objective measurements of heterophoria performed much better compared to standard clinical tests and thus, should become a valuable alternative in clinical practice.

### Ethics and Conflict of Interest

Each subject gave written informed consent before the experiments; the research followed the tenets of the Declaration of Helsinki and was approved by the Swiss ethics committee (https://www.swissethics.ch/; Project ID: 2017-01155). The authors declare no conflict of interest.

### Acknowledgements

This research was supported by a grant (320030_172965) from the Swiss National Science Foundation.
